# Supplementary material for: The causal relationship between the human gut microbiota and pyogenic arthritis: a Mendelian randomization study
Source: Front Cell Infect Microbiol. 2024 Nov 26;14:1452480. doi: 10.3389/fcimb.2024.1452480 (PMC11629706; doi:10.3389/fcimb.2024.1452480)
Supplement: Supplementary file 1 [file DataSheet1.zip › mendelian test/ebi-a-GCST90027790.csv_forest.pdf]

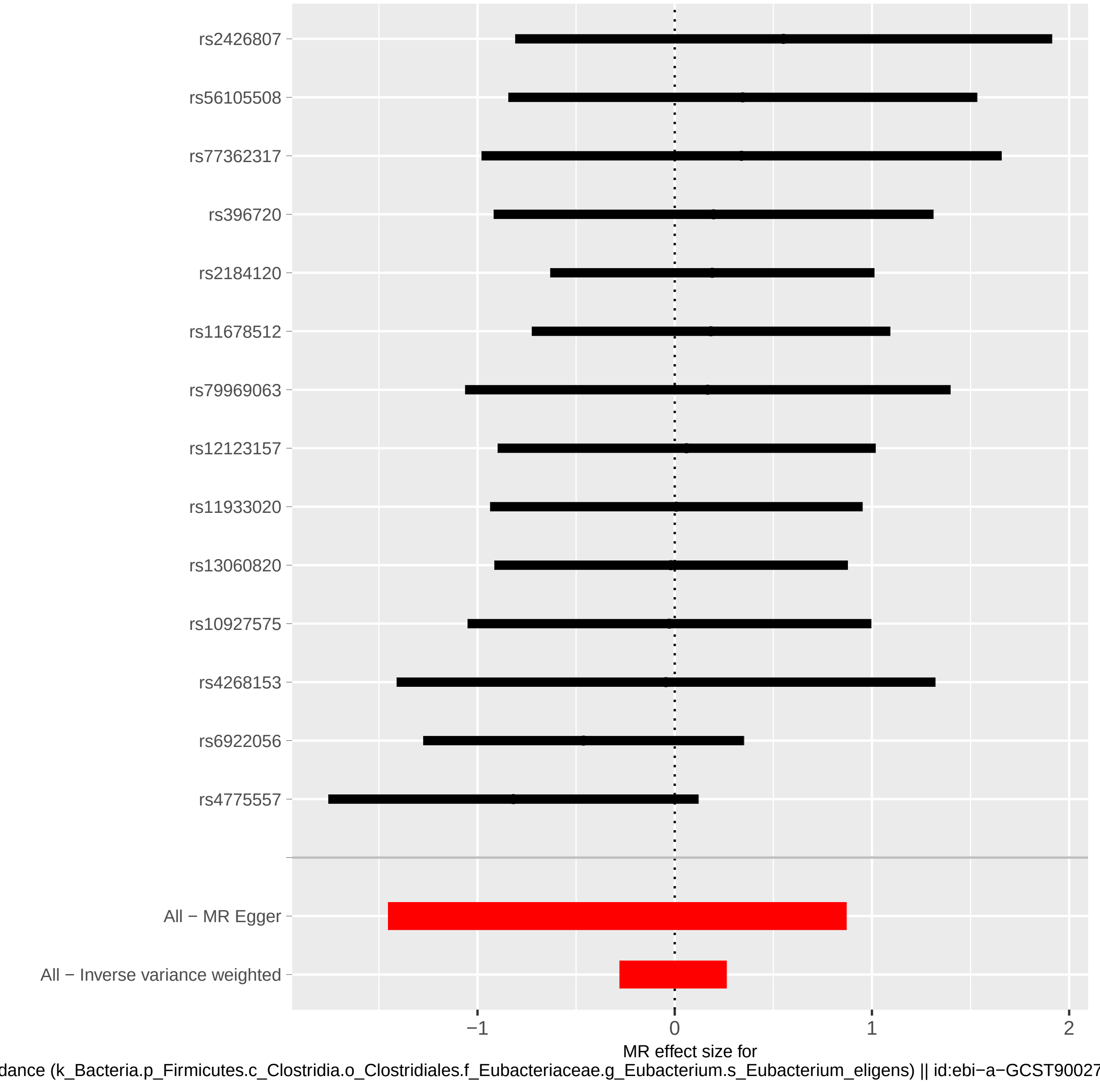

rs2426807
rs56105508
rs77362317
rs396720
rs2184120
rs11678512
rs79969063
rs12123157
rs11933020
rs13060820
rs10927575
rs4268153
rs6922056
rs4775557
All − MR Egger
All − Inverse variance weighted
−1
0
1
2
MR effect size for
dance (k_Bacteria.p_Firmicutes.c_Clostridia.o_Clostridiales.f_Eubacteriaceae.g_Eubacterium.s_Eubacterium_eligens) || id:ebi−a−GCST90027
